# Integrative transcriptomic and single-cell analysis reveals a convergent cholangiocyte-associated host inflammatory program in biliary atresia and SARS-CoV-2-associated hepatobiliary injury

**DOI:** 10.3389/fimmu.2026.1923630

**Published:** 2026-09-16

**Authors:** Yujuan Wa, Rong Guan, Zhanwei Du, Rouchen Lin, Shiyu Cui, Jianglei Guang, Lidan Xu, Wei Ji, Qian Yang, Qingbo Cui, Hao Wang, Wenjing Sun

**Affiliations:** 1 Department of Medical Genetics, School of Basic Medical Sciences, Harbin Medical University, Harbin, China; 2 Key Laboratory of Preservation of Human Genetic Resources and Disease Control in China (Harbin Medical University), Ministry of Education, Harbin, China; 3 Department of Microbiology, Harbin Medical University, Harbin, China; 4 Department of Pediatric Surgery, The Sixth Affiliated Hospital of Harbin Medical University, Harbin, China; 5 Department of Hepatopancreatobiliary Surgery, The Second Affiliated Hospital of Harbin Medical University, Harbin, China; 6 Laboratory of Medical Genetics, Heilongjiang Academy of Medical Sciences, Harbin, China

**Keywords:** biliary atresia, cholangiocyte, host inflammatory response, integrative transcriptomics, pediatric cholangiopathy, perinatal infection, SARS-CoV-2, single-cell transcriptomics

## Abstract

SARS-CoV-2-associated hepatobiliary injury and biliary atresia (BA) differ markedly in etiology and biological context, but both involve injury to the biliary epithelial compartment. Here, we performed integrative transcriptomic and single-cell analyses to investigate whether these distinct hepatobiliary injury contexts converge on a shared cholangiocyte-associated inflammatory host-response program. Bulk transcriptomic datasets from SARS-CoV-2-infected human liver organoids and BA liver tissues were integrated to identify shared transcriptional alterations, followed by functional enrichment, protein-protein interaction, regulatory network, drug-gene interaction, and molecular docking analyses. Single-nucleus and single-cell datasets were used to determine the cellular localization of the shared program, and key findings were further assessed in BA liver tissues and cultured human cholangiocytes exposed to poly(I:C). We identified an expanded exploratory set of 164 shared genes and a stringent *FDR*-adjusted core of 52 genes. Both sets consistently implicated antiviral defense, type I and type II interferon signaling, double-stranded RNA responses, and cytokine and chemokine signaling. STAT1, ISG15, and CXCL10 were retained in the stringent core and remained central components of the interaction network. In COVID-19 liver single-nucleus data, donor-level analysis showed preferential localization of the shared signature to cholangiocytes, whose mean scores were significantly higher than those of all other hepatic cells combined (*P* = 0.00368). A similar pattern was observed descriptively in our BA single-nucleus dataset and further supported by an independent BA single-cell cohort. Tissue immunofluorescence spatially associated pSTAT1, total STAT1, and ISG15 signals with KRT19-positive ductular structures, whereas CXCL10 was predominantly periductal. Poly(I:C) stimulation induced representative components of the program in cultured cholangiocytes. Computational analyses retained mefloquine among the top-ranked exploratory candidates. Collectively, these findings support a convergent cholangiocyte-associated interferon/inflammatory host-response program in BA and SARS-CoV-2-associated hepatobiliary injury. They indicate molecular association rather than a common etiology or causal relationship and provide testable hypotheses for further investigation of biliary epithelial inflammatory responses.

## 1 Introduction

Following the global COVID-19 public health emergency ending in May 2023, researchers have increasingly focused on understanding the long-term interaction between humans and Severe Acute Respiratory Syndrome Coronavirus 2 (SARS-CoV-2) and its potential impacts on multiple organ systems. Hepatobiliary injury is increasingly recognized as an extrapulmonary manifestation of viral diseases, including coronavirus disease 2019 (COVID-19). Although the respiratory tract is the principal target of SARS-CoV-2 infection, clinical and pathological studies have shown that the liver and biliary system may also be affected in a subset of patients (1, 2). This extrapulmonary involvement is partly related to the broad tissue distribution of angiotensin-converting enzyme 2 (ACE2), the major entry receptor for SARS-CoV-2, and the reported expression of ACE2 in biliary epithelial cells (3–5). Although ACE2 expression alone does not establish productive viral infection or direct cholangiocyte injury, these findings suggest that cholangiocytes may represent a vulnerable epithelial compartment during virus-associated hepatobiliary injury. However, the host-response programs associated with cholangiocyte involvement during SARS-CoV-2-associated hepatobiliary injury remain incompletely defined.

Biliary atresia (BA) is a rare but life-threatening neonatal cholangiopathy marked by progressive inflammation and fibrotic obstruction of the bile ducts (6). Approximately 80% of patients are categorized as having the perinatal or acquired form of BA (7). BA is etiologically heterogeneous, and although its precise etiology remains unresolved, perinatal immune dysregulation, viral exposure, and environmental factors have long been implicated as potential contributors to bile duct injury. Several viruses, including rotavirus and cytomegalovirus, have been associated with immune-mediated biliary epithelial injury in BA-related studies (7, 8). However, these associations are not consistently observed across patient populations and do not establish viral infection as a universal cause of human BA. Therefore, BA provides a clinically relevant pediatric cholangiopathy context in which to investigate how distinct infectious or inflammatory insults may converge on cholangiocyte-associated host-response programs, without presupposing a common etiology with SARS-CoV-2-associated hepatobiliary injury.

Recent advances in organoid modeling, bulk transcriptomics, and single-cell sequencing provide new opportunities to compare host-response programs across distinct disease contexts. Despite substantial differences in patient age, cellular composition, tissue architecture, immune-cell content, and disease stage, the integration of publicly available bulk and single-cell datasets with in-house BA single-nucleus sequencing makes it possible to examine whether virus-associated hepatobiliary injury and pediatric cholangiopathy share molecular features at both transcriptomic and cellular-resolution levels. Such cross-disease analyses do not establish direct etiological causality between SARS-CoV-2 infection and BA or demonstrate that the identified responses are specific to either disease. Rather, they can determine whether biologically distinct injury contexts converge on recurrent inflammatory programs within the biliary epithelial compartment and generate testable hypotheses for subsequent mechanistic investigation.

In this study, we performed integrative transcriptomic and single-cell analyses to investigate whether SARS-CoV-2-associated hepatobiliary injury and BA converge on a cholangiocyte-associated inflammatory host-response program. Bulk RNA-sequencing datasets from SARS-CoV-2-infected human liver organoids and BA liver tissues were integrated to identify overlapping transcriptional alterations, including an expanded exploratory gene set and a stringent *FDR*-adjusted core, followed by functional enrichment and protein-protein interaction analyses. COVID-19 liver single-nucleus transcriptomic data were analyzed to define the cellular localization of the shared signature and its variation across inferred cholangiocyte states. BA liver single-nucleus sequencing and an independent BA single-cell dataset were used to provide descriptive supporting evidence and independent corroboration, respectively. Key findings were further assessed in BA liver tissues and cultured human cholangiocytes exposed to poly(I:C), and candidate compounds were computationally prioritized through drug-gene interaction and molecular docking analyses. Through this multilayered approach, we sought to define a systems-level framework for convergent biliary epithelial host responses and to generate testable biological and exploratory therapeutic hypotheses, rather than to establish a common etiology, diagnostic performance, or therapeutic efficacy. The overall analytical workflow is shown in **Figure 1A**.

**Figure 1:**
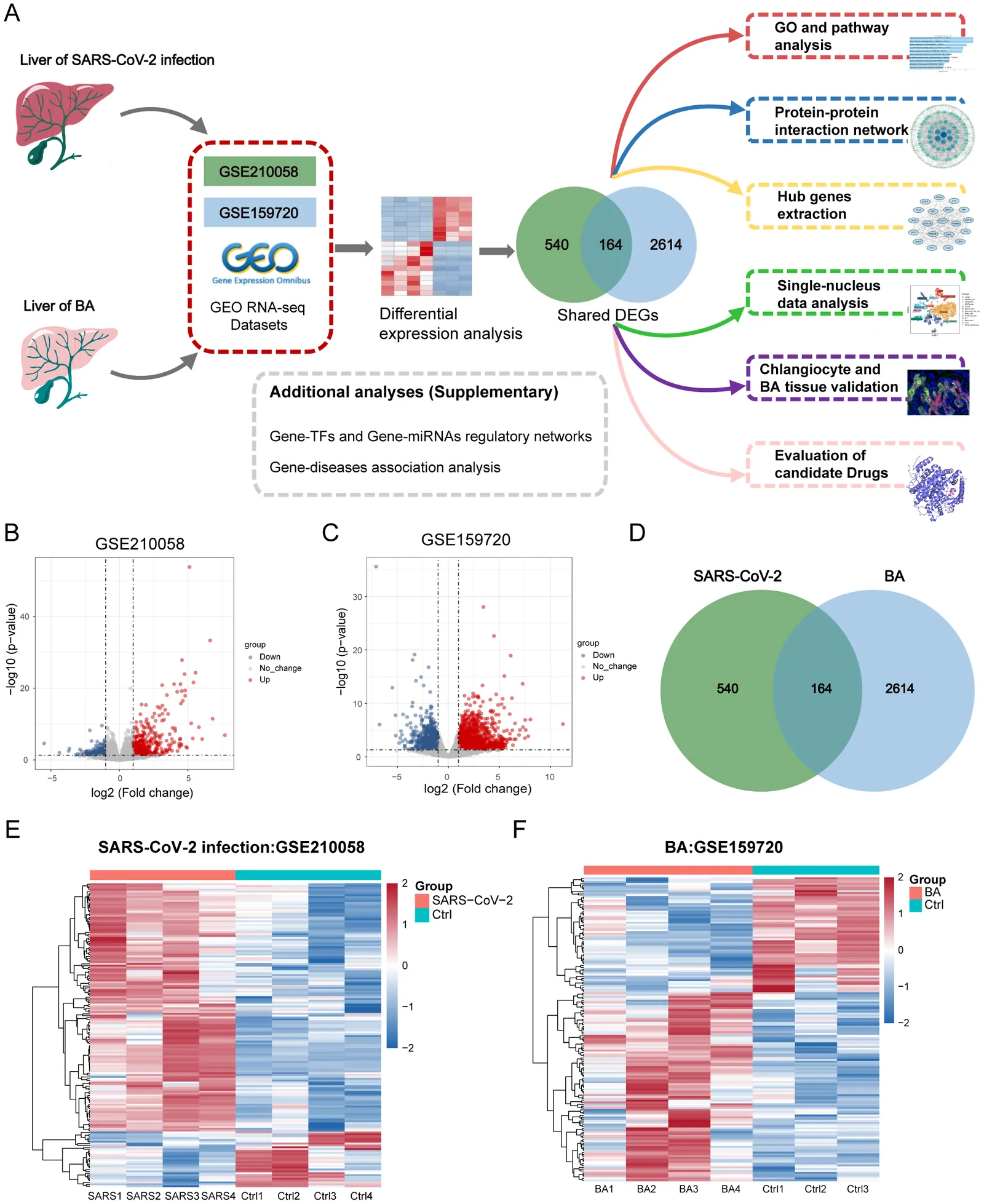
Identification of shared transcriptional features between SARS-CoV-2-associated hepatobiliary injury and BA. **(A)** Overall study workflow. **(B, C)** Volcano plots exhibit differentially expressed genes (DEGs) of SARS-CoV-2 infection and biliary atresia, with |log_2_FC| > 1 and *P*-value < 0.05. **(D)** The Venn diagram describes the DEGs shared between SARS-CoV-2 infection and biliary atresia. **(E, F)** Heatmap of the 164 shared DEGs illustrating common transcriptional alterations between SARS-CoV-2 associated hepatobiliary injury and BA. Expression values were log_2_-transformed and scaled by gene using row-wise Z scores. Colors therefore represent the relative expression of each gene across samples rather than absolute expression levels or binary pathway activation.

## 2 Materials and methods

### 2.1 Patient samples

Liver tissues were collected from four patients with biliary atresia (BA) and one patient with choledochal cyst (CC) at the Sixth Affiliated Hospital of Harbin Medical University. Paraffin-embedded liver tissue from one BA patient was used for single-nucleus RNA sequencing (snRNA-seq), whereas samples from the other three BA patients and the CC control were subjected to immunofluorescence (IF) analysis. The Ethics Committee of Harbin Medical University approved this study (No. HMUIRB2024036), and informed consent was obtained for all research individuals.

### 2.2 Datasets acquisition

In order to identify the shared genetic links between SARS-CoV-2 infection and BA, two RNA-sequencing datasets were downloaded from the GEO database of the NCBI (http://www.ncbi.nlm.nih.gov/geo/). The SARS-CoV-2 infection dataset was obtained from the GEO database under accession number GSE210058 (9). The BA dataset was obtained under accession number GSE159720 (10). Raw snRNA-seq data from COVID-19 liver tissues were retrieved from GSE171668 (11). Raw scRNA-seq data from BA and CC liver tissues were retrieved from GSE176189 (12).

### 2.3 Differentially expressed gene analysis and mutual DEGs identification

Differential expression analysis for SARS-CoV-2 infection (GSE210058) and BA (GSE159720) was performed using the DESeq2 *R* package. Differentially expressed genes with an absolute log_2_ fold change > 1 and a Benjamini-Hochberg-adjusted *P* value < 0.05 were defined as the stringent *FDR*-adjusted gene sets. In parallel, genes meeting the same fold-change criterion (|log_2_ fold change| > 1) but a nominal *P* value < 0.05 were retained as an expanded exploratory set for visualization and hypothesis-generating analyses. Jvenn (http://jvenn.toulouse.inra.fr/app/index.html), a VENN diagram viewing tool, provided the shared DEGs of GSE210058 and GSE159720 (13).

### 2.4 Gene ontology and pathway enrichment analysis

To investigate the potential biological mechanisms underlying the shared DEGs between SARS-CoV-2 infection and BA, we performed functional enrichment analyses, including GO annotation and pathway enrichment, using Enrichr (https://maayanlab.cloud/Enrichr/), with *P* value < 0.05.

### 2.5 Protein-protein interaction network analysis, submodule analysis and hub gene extraction

STRING database (https://www.string-db.org/) (version 11.5) was applied to construct PPI networks for the shared DEGs (14, 15). For the creation of the PPI network of the mutual DEGs, we set a confidence score > 0.4, and imported the visual results into Cytoscape (v.3.9.1). We applied the Molecular Complex Detection (MCODE) algorithm within Cytoscape to detect gene modules in the PPI network. Furthermore, we used a novel Cytoscape plugin, cytoHubba (http://apps.cytoscape.org/apps/cytohubba), for ranking the importance of nodes in a network according to their network features. Using Cytohubba’s Maximum Neighborhood Component (MNC) algorithm, we identified the 20 most central hub genes in the PPI network (14).

### 2.6 Single-nucleus RNA sequencing of BA

Single-nucleus RNA sequencing was performed by SeekGene (Beijing, China) using the Illumina NovaSeq 6000 platform. Single-nucleus suspensions were prepared from FFPE liver tissue of BA using the SeekMate FFPE Dissociation Kit. Briefly, 50 μm sections were deparaffinized, rehydrated, and homogenized, and nuclei were counted using a fluorescence cell analyzer (Countstar^®^ Rigel S2). Following nuclear RNA extraction using the SeekOne^®^ DD Single Cell Decrosslinking Kit, qualified nuclei were adjusted to a concentration of 1500~2000 nuclei/μL.

Following the manufacturer’s protocol, we used the SeekOne^®^ DD FFPE Single Cell Transcriptome-seq Kit (SeekGene) to generate single-nucleus RNA-seq libraries. Briefly, nuclei underwent reverse transcription, droplet encapsulation with barcoded hydrogel beads using the SeekOne^®^ Digital Droplet System, followed by cDNA amplification and library construction. The final libraries underwent purification and quality control, followed by 150 bp paired-end sequencing on the Illumina NovaSeq 6000 platform.

### 2.7 Analysis of single-nucleus and single-cell data

Raw snRNA-seq and scRNA-seq data were obtained and raw 10x Genomics HDF5 files were processed using the Seurat package (v5.0) to build Seurat objects. Quality control was applied to remove low-quality nuclei. Cells with fewer than 200 or more than 6000 detected genes were excluded, and nuclei with mitochondrial transcript proportions above 20% were also discarded. DoubletFinder (v2.0.4) was then used to detect and eliminate potential doublets. To reduce sample-to-sample variability, batch integration was carried out using the Harmony package (v1.2.1). Subsequent analyses were conducted in Seurat, where the data were normalized, highly variable genes were selected, and the expression matrix was scaled. Principal component analysis was performed prior to Harmony-based correction and graph-based clustering. Cell type annotation was performed based on canonical marker genes using the CellMarker 2.0 database (http://xteam.xbio.top/CellMarker/) as a reference.

A gene set comprising the expanded 164-gene shared signature was used to calculate module scores at the single-nucleus level using the AddModuleScore function in Seurat. For visualization, module scores were transformed to a 0–1 range across the complete dataset, whereas the unscaled module scores were used for donor-level statistical analyses. To avoid pseudoreplication, individual nuclei were not treated as independent biological replicates. For comparisons across major hepatic cell populations, nucleus-level scores were averaged for each cell type within each donor, with each donor treated as an independent biological replicate. For the paired comparison, the mean signature score was calculated separately for cholangiocytes and for all other hepatic cells combined within each donor. Donor-matched values were compared using a two-sided paired *Wilcoxon* signed-rank test. For the biliary epithelial cell subtype analysis, module scores were averaged for each BEC subtype within each donor, and each point represents one donor-subtype combination. Because of the limited number of donors and unequal representation of BEC subtypes across specimens, this analysis was interpreted descriptively without inferential testing. The in-house BA single-nucleus dataset, which comprised one BA specimen, was used only for descriptive cellular-localization analysis, and no inferential statistical testing was performed. After isolating cholangiocytes from the independent single-cell transcriptomic dataset according to cell annotation, differential gene expression between the BA and CC groups was analyzed using the Seurat FindMarkers function.

### 2.8 Pseudotime trajectory analysis

We performed cellular trajectory analysis using the Monocle2 package (version 2.24.0). The Seurat object was first converted into a Monocle CellDataSet using the raw count matrix together with cell metadata and gene annotation information. We then applied the DDRTree algorithm for dimensionality reduction and ordered cells along pseudotime using the orderCells function. Gene expression patterns along the inferred pseudotime trajectory were visualized.

### 2.9 Cell culture and Poly(I:C) stimulation

The human cholangiocyte cell line H69 was kindly provided by Dr. Nidhi Jalan Sakrikar (Mayo Clinic, Rochester, USA). The H69 cholangiocytes were cultured in H69 media-DMEM/F12 (Gibco) supplemented with 10% FBS, insulin, epinephrine, hydrocortisone and 1% penicillin-streptomycin. For viral-mimetic stimulation, cells were treated with polyinosinic-polycytidylic acid [Poly(I:C); 30 μg/mL; MedChemExpress, Cat. No. HY-107202; CAS No. 24939-03-5] for 24 h. Untreated cells cultured in parallel for the same duration served as controls. Cells were subsequently harvested for qRT-PCR and Western blot analyses.

### 2.10 Immunofluorescence staining

Frozen liver sections (6~8 μm) were fixed in 4% paraformaldehyde, permeabilized with 0.1% Triton X-100, and blocked with 5% bovine serum albumin. The sections were incubated overnight at 4°C with primary antibodies against KRT19 (Thermo Fisher Scientific), STAT1, pSTAT1, CXCL10 and ISG15 (all from Proteintech). After washing, appropriate fluorescent secondary antibodies (Thermo Fisher Scientific) were applied for 1 h at room temperature. Nuclei were counterstained with DAPI, and fluorescence images were captured using a confocal microscope.

### 2.11 Quantitative reverse transcription PCR and Western blot

Total RNA was extracted from samples using TRIzol reagent (Invitrogen) following the manufacturer’s protocol. The concentration and purity of RNA were determined prior to reverse transcription. For cDNA synthesis, 2 μg of total RNA was reverse-transcribed in a 20 μL reaction volume using the Transcriptor First Strand cDNA Synthesis Kit (Roche). Quantitative real-time PCR was subsequently performed using LightCycler^®^ 480 SYBR Green I Master Mix (Roche) on a LightCycler 480 Real-Time PCR system. Relative gene expression levels were calculated using the 2^-ΔΔCt method after normalization to the internal control gene. The primer sequences used in this study are listed in **Supplementary Table 1**.

For Western blot, cells were lysed in RIPA buffer supplemented with protease and phosphatase inhibitors. Equal amounts of protein were separated by SDS-PAGE, transferred onto PVDF membranes, and incubated with the following primary antibodies overnight at 4 °C: phospho-STAT1 (Tyr701) (Proteintech, 28979-1-AP), STAT1 (Proteintech, 10144-2-AP), ISG15 (Proteintech, 15981-1-AP), and CXCL10 (Proteintech, 85307-2-RR). After incubation with the corresponding secondary antibodies, protein bands were visualized using enhanced chemiluminescence. Band intensities were quantified using ImageJ, with phospho-STAT1 normalized to total STAT1 and the remaining proteins normalized to the loading control.

### 2.12 Transcription factors and miRNAs identification

We discovered the DEG-transcription factors (TFs) and the DEG-miRNA interaction network in order to identify the major regulators and gain a better understanding of the most significant transcriptional alterations. To find reliable TFs from the JASPAR database and miRNAs from the Tarbase and mirTarbase, we utilized the NetworkAnalyst software (16, 17), with a degree cutoff = 2, and based on topological analysis (14, 15, 18, 19).

### 2.13 Gene-disease association analysis

With the most detailed and public catalog of genes and genomic variants associated with human diseases, DisGeNET is a knowledge management platform. Its core concepts are Gene-Disease Association (GDA) and Variant-Disease Association (VDA) (15). The gene-disease association network has been created using the NetworkAnalyst platform (with a degree cutoff = 2) to reveal related diseases and their chronic complications associated with common DEGs (14, 15).

### 2.14 Evaluation of applicant drugs

The shared DEGs between SARS-CoV-2 infection and BA were used in this study to predict pharmacological compounds and protein-drug interactions (PDIs). Enrichr is a popular method for analyzing gene sets generated from whole-genome experiments. DSigDB is a special gene set collection based on quantitative inhibition and/or drug-induced gene expression change data of drugs and chemicals. Enrichr’s Disease/Drugs function is one approach to access the DSigDB database (14, 20).

### 2.15 Molecular docking

Molecular docking was used to evaluate the interactions between candidate compounds and key SARS-CoV-2 related targets. Based on previous literature, five representative viral or host proteins associated with SARS-CoV-2 infection were selected, including ACE2 (angiotensin-converting enzyme 2), 3CLpro (3C-Like protease), Mpro (main protease), PLpro (papain-like protease) and RdRp (RNA-dependent RNA polymerase) (21). For subsequent molecular docking analysis, the crystal structures of these key proteins were downloaded from the Protein Data Bank (PDB) (https://www.rcsb.org/) (14). These five important proteins’ PDB codes are as follows: ACE2 is 1R42, 3CLpro is 6LU7, Mpro is 6WUU, PLpro is 6Y2E, and RdRp is 7BV2. The chemical structures of candidate compounds were collected from the PubChem database (https://pubchem.ncbi.nlm.nih.gov/) and subsequently subjected to molecular docking using AutoDock Vina implemented in Chimera (v1.16). Protein and ligand structures were prepared prior to docking, and grid box parameters were defined to encompass the active binding sites. Docking was conducted in rigid mode between the receptor and ligand. The binding pose with the lowest binding energy was selected as the optimal conformation and further visualized and analyzed using Discovery Studio 4.5 Client.

In addition, to strengthen the connection between the molecular docking analysis and the BA-related viral context, two additional viral proteins previously implicated in BA research were included. These comprised the human cytomegalovirus UL80 protease (PDB ID: 8J3T) and a structurally characterized rotavirus VP1 RNA-dependent RNA polymerase (PDB ID: 2R7X). The corresponding protein structures were obtained from the Protein Data Bank and prepared using the same procedure described above. Mefloquine was docked against these two viral proteins using AutoDock Vina implemented in Chimera (v1.16), and the binding pose with the lowest predicted binding energy was selected as the optimal conformation for subsequent visualization and analysis using Discovery Studio 4.5 Client.

### 2.16 Statistical analysis

All statistical analyses were mainly conducted in the *R* environment (version 4.1.3). Differential expression analyses for RNA-seq data were carried out using the DESeq2 package, with Benjamini-Hochberg-adjusted *P* values used for multiple-testing correction. Processing and visualization of snRNA and scRNA sequencing data were performed using commonly used *R* packages, including Seurat and ggplot2. qRT-PCR and Western blot data were obtained from three independent biological experiments and results were analyzed in GraphPad Prism (version 9.0) using Student’s *t*-test. A two-tailed *P* value < 0.05 was considered statistically significant.

## 3 Results

### 3.1 Identification of shared differentially expressed genes between SARS-CoV-2 associated hepatobiliary injury and biliary atresia

To explore transcriptional similarities between SARS-CoV-2 associated liver injury and biliary atresia (BA), transcriptomic data were analyzed. In the SARS-CoV-2 dataset (GSE210058), 704 differentially expressed genes (DEGs) were identified, including 462 upregulated and 242 downregulated genes. In the BA dataset (GSE159720), 2778 DEGs were detected, comprising 1628 upregulated and 1150 downregulated genes. Detailed dataset information is summarized in **Table 1**. Volcano plots illustrate the overall transcriptional changes in SARS-CoV-2-infected liver organoids and BA liver tissues (**Figures 1B, C**).

**Table 1:** Overview of the transcriptomic datasets used in this study.

Disease name	GEO accession	GEO platform	Total DEGs count	Up regulated DEGs count	Down regulated DEGs count
SARS-CoV-2	GSE210058	GPL24676	704	462	242
BA	GSE159720	GPL18573	2778	1628	1150

Intersection analysis using Jvenn identified 164 genes shared between the two exploratory DEG sets (**Figure 1D**), and the complete gene list is provided in **Supplementary Table 2**. These shared genes indicate a measurable transcriptional overlap between SARS-CoV-2-associated hepatobiliary injury and BA. To visualize the distribution of the expanded shared gene set across individual samples, heatmaps showing the expression of all 164 shared genes in the SARS-CoV-2-infected liver organoid and BA liver datasets are presented in **Figures 1E, F**, respectively. The heatmaps revealed recurrent but heterogeneous expression patterns across the included samples, indicating that the shared transcriptional program was not uniformly represented in every individual specimen.

To assess whether this transcriptional overlap remained detectable after multiple-testing correction, differential-expression analyses were repeated using the same fold-change threshold together with a Benjamini-Hochberg-adjusted *P* value < 0.05. This stringent analysis identified 315 DEGs in the SARS-CoV-2-infected liver organoid dataset and 1485 DEGs in the BA dataset, with 52 genes shared between the two datasets. All 52 genes were contained within the expanded 164-gene set, thereby defining a stringent *FDR*-adjusted core. The *FDR*-adjusted DEG overlap and the corresponding analyses are presented in **Supplementary Figure 2A**.

### 3.2 Integrated enrichment and network analyses define a shared interferon-dominant host inflammatory program

To characterize the biological significance of the expanded 164-gene shared set, Gene Ontology (GO) enrichment analysis was performed (**Figure 2A**). The top 10 enriched terms in biological process (BP), molecular function (MF), and cellular component (CC) are summarized in **Table 2**. BP analysis showed that these genes were mainly involved in antiviral and immune-related processes, including defense responses to virus and symbiont, as well as cellular responses to type I interferon. In the MF category, the enriched terms included growth factor receptor binding, receptor ligand activity, CXCR chemokine receptor binding, and double-stranded RNA binding, suggesting participation in inflammatory and immune signaling. In the CC category, the expanded 164-gene shared set was primarily associated with the cell surface and membrane-related structures, including the actin cytoskeleton, brush border membrane, motile cilium, and microvillus.

**Figure 2:**
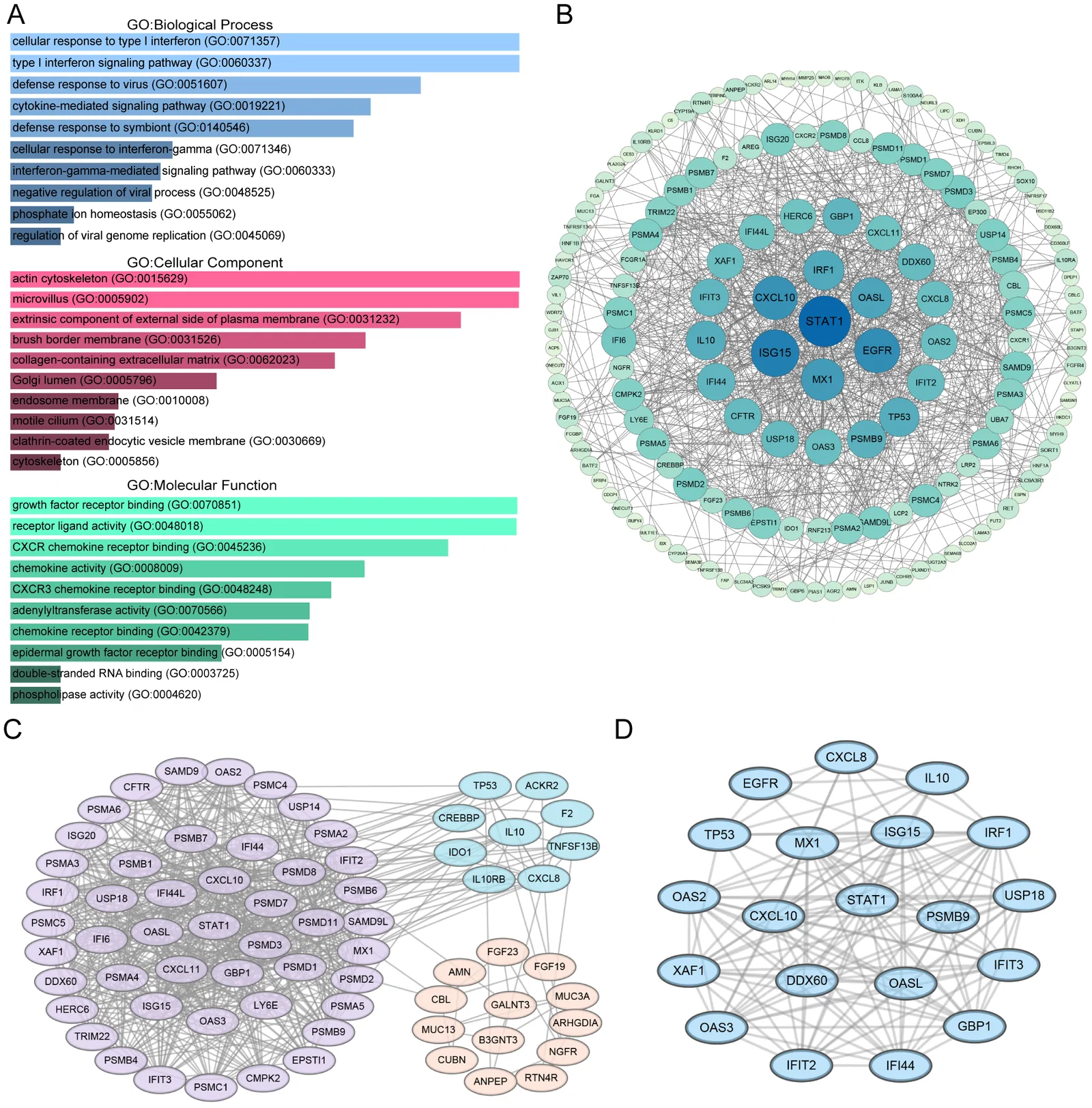
Functional enrichment and network analyses identify a STAT1-centered shared inflammatory program. **(A)** Functional enrichment analysis of the 164 shared DEGs. **(B)** The PPI network of the shared DEGs between SARS-CoV-2 infection and biliary atresia, and DEGs as circle nodes and interactions between nodes as edges. There are 924 edges and 160 nodes in the PPI network with the PPI enrichment *P*-value < 0.001. **(C)** The first three modules of genes in the PPI network were found by MCODE. **(D)** The top 20 hub genes from the PPI network were found using the MNC process of the Cytohubba plugin in Cytoscape.

**Table 2:** Ontological analysis of the expanded 164-gene shared set between SARS-CoV-2 infection and BA.

Category	GO ID	Term	*P*-values	Genes
BP	GO:0071357	cellular response to type I interferon	4.9079E-12	ISG20, OAS2, STAT1, OAS3, MX1, IFI6, ISG15, XAF1, IFIT3, IFIT2, OASL
	GO:0060337	type I interferon signaling pathway	4.9079E-12	ISG20, OAS2, STAT1, OAS3, MX1, IFI6, ISG15, XAF1, IFIT3, IFIT2, OASL
	GO:0051607	defense response to virus	7.1577E-11	STAT1, MX1, IFI6, ISG15, IFI44L, IFIT3, IFIT2, OASL, ISG20, SLFN13, CXCL10, OAS2, OAS3
	GO:0019221	cytokine-mediated signaling pathway	2.7897E-10	IL10, CXCL8, STAT1, MX1, IFI6, ISG15, IFIT3, BATF, PSMB9, IFIT2, OASL, TNFSF13B, ISG20, CXCL10, CXCL11, CCL8, OAS2, OAS3, XAF1, CD300LF, TRIM31, FCGR1A, GBP1, TRIM22
	GO:0140546	defense response to symbiont	4.4408E-10	ISG20, SLFN13, OAS2, STAT1, OAS3, MX1, IFI6, ISG15, IFI44L, IFIT3, IFIT2, OASL
	GO:0071346	cellular response to interferon-gamma	6.042E-08	GBP6, CCL8, OAS2, STAT1, OAS3, FCGR1A, TRIM31, GBP1, TRIM22, OASL
	GO:0060333	interferon-gamma-mediated signaling pathway	8.3828E-08	OAS2, STAT1, OAS3, FCGR1A, TRIM31, GBP1, TRIM22, OASL
	GO:0048525	negative regulation of viral process	1.0558E-07	ISG20, OAS2, STAT1, OAS3, MX1, ISG15, TRIM31, OASL
	GO:0055062	phosphate ion homeostasis	8.8072E-07	SFRP4, SLC34A2, FGFR4, FGF23
	GO:0045069	regulation of viral genome replication	1.2612E-06	ISG20, CXCL8, OAS2, OAS3, MX1, ISG15, OASL
MF	GO:0070851	growth factor receptor binding	0.00022854	IL10, AGR2, CBLC, CD300LF, FGF23, AREG
	GO:0048018	receptor ligand activity	0.00022966	SEMA6B, IL10, CXCL10, COLEC10, GDF2, SEMA3E, F2, FGF23, AREG, TNFSF13B
	GO:0045236	CXCR chemokine receptor binding	0.00033828	CXCL10, CXCL11, CXCL8
	GO:0008009	chemokine activity	0.00054384	CXCL10, CXCL11, CXCL8, CCL8
	GO:0048248	CXCR3 chemokine receptor binding	0.00065755	CXCL10, CXCL11
	GO:0070566	adenylyltransferase activity	0.0007434	OAS2, OAS3, OASL
	GO:0042379	chemokine receptor binding	0.00074825	CXCL10, CXCL11, CXCL8, CCL8
	GO:0005154	epidermal growth factor receptor binding	0.00122527	AGR2, CBLC, AREG
	GO:0003725	double-stranded RNA binding	0.00305695	OAS2, OAS3, DDX60, OASL
	GO:0004620	phospholipase activity	0.00305695	LIPC, PITPNM3, ASPG, PLA2G2A
CC	GO:0015629	actin cytoskeleton	0.00122183	VIL1, CLDN5, ONECUT2, MYO7B, DCDC2, ACKR2, CAPG, LSP1, GBP1
	GO:0005902	microvillus	0.00122763	VIL1, MYO7B, CDHR5, ESPN
	GO:0031232	extrinsic component of external side of plasma membrane	0.00181157	CUBN, PCSK9
	GO:0031526	brush border membrane	0.00342809	CUBN, SLC34A2, CDHR5
	GO:0062023	collagen-containing extracellular matrix	0.00421274	ITIH5, TINAGL1, TNN, ANGPTL6, LAMA1, MMRN2, LAMA3, S100A4, F2
	GO:0005796	Golgi lumen	0.00929233	MUC13, F2, MUC3A, FGF23
	GO:0010008	endosome membrane	0.0179353	RET, CUBN, AMN, LAMP3, PCSK9, FCGR1A, CFTR
	GO:0031514	motile cilium	0.0183905	DNAH11, DNAH9, HAVCR1
	GO:0030669	clathrin-coated endocytic vesicle membrane	0.01911325	FCGR1A, AREG, CFTR
	GO:0005856	cytoskeleton	0.02639251	VIL1, ONECUT2, MX1, MYO7B, DCDC2, RHOH, CAPG, LSP1, DDX60, GBP1

To further define the signaling context of the expanded 164-gene shared set, pathway enrichment analysis was performed using the Reactome, BioPlanet, WikiPathways, and KEGG databases (**Supplementary Figure 1**), and the top enriched pathways are listed in **Table 3**. Across these databases, the expanded 164-gene shared set was consistently enriched in interferon, immune, and infection related pathways, including interferon signaling, SARS-CoV-2 innate immune evasion, coronavirus disease pathways, and viral protein interaction with cytokines.

**Table 3:** Pathway enrichment analysis of common DEGs between SARS-CoV-2 infection and BA.

Category	Pathways	*P*-values	Genes
Reactome 2022	Interferon Signaling R-HSA-913531	9.19228E-12	GBP6, STAT1, MX1, IFI6, ISG15, IFIT3, IFIT2, OASL, ISG20, OAS2, OAS3, XAF1, TRIM31, FCGR1A, GBP1, TRIM22
	Interferon Alpha/Beta Signaling R-HSA-909733	1.57756E-11	ISG20, OAS2, STAT1, OAS3, MX1, IFI6, ISG15, XAF1, IFIT3, IFIT2, OASL
	Interferon Gamma Signaling R-HSA-877300	4.87751E-08	GBP6, OAS2, STAT1, OAS3, FCGR1A, TRIM31, GBP1, TRIM22, OASL
	Cytokine Signaling In Immune System R-HSA-1280215	7.16543E-08	GBP6, IL10, CXCL8, STAT1, MX1, IFI6, ISG15, IFIT3, BATF, PSMB9, IFIT2, OASL, TNFSF13B, ISG20, CXCL10, OAS2, OAS3, XAF1, TRIM31, FCGR1A, GBP1, TRIM22
	Plasma Lipoprotein Clearance R-HSA-8964043	9.27869E-06	CUBN, AMN, LIPC, PCSK9, CES3
	Immune System R-HSA-168256	1.63829E-05	ITK, COLEC10, CXCL8, TINAGL1, C5AR2, IFI6, IFIT3, IFIT2, OASL, TNFSF13B, RNF213, ANPEP, MUC13, CD300LF, FCGR1A, GBP1, XDH, TRIM22, HERC6, GBP6, IL10, STAT1, MX1, PLA2G2A, ISG15, BATF, PSMB9, ISG20, CXCL10, OAS2, OAS3, KLRD1, XAF1, TRIM31
	OAS Antiviral Response R-HSA-8983711	4.38543E-05	OAS2, OAS3, OASL
	Antiviral Mechanism By IFN-stimulated Genes R-HSA-1169410	6.21569E-05	OAS2, STAT1, OAS3, MX1, ISG15, OASL
	Plasma Lipoprotein Assembly, Remodeling, And Clearance R-HSA-174824	0.000293451	CUBN, AMN, LIPC, PCSK9, CES3
	Biological Oxidations R-HSA-211859	0.000446784	CYP26A1, GLYATL1, MAOB, SULT1E1, DPEP1, UGT2A3, CES3, CYP19A1
BioPlanet 2019	Interferon alpha/beta signaling	4.11E-12	ISG20, OAS2, STAT1, OAS3, MX1, IFI6, ISG15, XAF1, IFIT3, IFIT2, OASL
	Interferon signaling	1.12E-10	GBP6, STAT1, MX1, IFI6, ISG15, IFIT3, IFIT2, OASL, ISG20, OAS2, OAS3, XAF1, FCGR1A, GBP1
	Immune system signaling by interferons, interleukins, prolactin, and growth hormones	8.13E-08	GBP6, STAT1, MX1, IFI6, ISG15, IFIT3, IFIT2, OASL, ISG20, OAS2, OAS3, XAF1, FCGR1A, GBP1
	Type II interferon signaling (interferon-gamma)	1.63E-07	CXCL10, STAT1, IFI6, ISG15, GBP1, IFIT2, PSMB9
	Interferon-gamma signaling pathway	1.52E-05	GBP6, OAS2, STAT1, OAS3, FCGR1A, GBP1, OASL
	Thymic stromal lymphopoietin (TSLP) pathway	9.78E-05	IL10, CXCL10, CXCL8, LAMP3, IDO1, PSMB9
	Interleukin-7 interactions in immune response	0.000166829	CXCL10, CXCL11, CXCL8, CCL8
	T cell receptor regulation of apoptosis	0.000460004	IL10, CXCL8, STAT1, MX1, RHOH, LSP1, F2, PSMB9, CXCL10, CXCL11, KLRD1, S100A4, TRIM31, GBP1
	Klotho-mediated ligand binding	0.000981016	FGFR4, FGF23
	Binding of chemokines to chemokine receptors	0.001001742	CXCL10, CXCL11, CXCL8, ACKR2
WikiPathway 2021 Human	Type II interferon signaling (IFNG) WP619	1.83496E-08	CXCL10, STAT1, IFI6, ISG15, GBP1, IFIT2, PSMB9
	SARS-CoV-2 innate immunity evasion and cell-specific immune response WP5039	1.13725E-06	IL10, CXCL10, CXCL11, CXCL8, STAT1, MX1, IFIT2
	COVID-19 adverse outcome pathway WP4891	0.000229096	IL10, CXCL10, CXCL8
	Non-genomic actions of 1,25 dihydroxyvitamin D3 WP4341	0.000293451	CXCL8, OAS2, STAT1, ISG15, IFI44L
	Tryptophan metabolism WP465	0.00038264	MAOB, AOX1, CYP19A1, IDO1
	Allograft Rejection WP2328	0.000831192	IL10, CXCL11, CXCL8, C6, STAT1
	Immune response to tuberculosis WP4197	0.000849779	STAT1, MX1, IFIT3
	Molybdenum cofactor (Moco) biosynthesis WP4507	0.001366028	AOX1, XDH
	Effects of nitric oxide WP1995	0.001811568	AOX1, XDH
	Type I interferon induction and signaling during SARS-CoV-2 infection WP4868	0.002055706	STAT1, OAS2, OAS3
KEGG 2021 Human	Coronavirus disease	0.000129938	CXCL10, C6, CXCL8, OAS2, STAT1, OAS3, MX1, ISG15, F2
	Maturity onset diabetes of the young	0.001225272	ONECUT1, HNF1B, HNF1A
	Viral protein interaction with cytokine and cytokine receptor	0.00140279	IL10, CXCL10, CXCL11, CXCL8, CCL8
	Steroid hormone biosynthesis	0.001581245	HSD11B2, SULT1E1, UGT2A3, CYP19A1
	Hepatitis C	0.00189263	CLDN5, CXCL10, OAS2, STAT1, OAS3, MX1
	Influenza A	0.002985934	CXCL10, CXCL8, OAS2, STAT1, OAS3, MX1
	Cytokine-cytokine receptor interaction	0.003065584	IL10, CXCL10, NGFR, CXCL11, CXCL8, CCL8, GDF2, TNFSF13B
	NOD-like receptor signaling pathway	0.003835942	NLRP12, CXCL8, OAS2, STAT1, OAS3, GBP1
	Tryptophan metabolism	0.004915868	MAOB, AOX1, IDO1
	Chemokine signaling pathway	0.005104338	CXCL10, ITK, CXCL11, CXCL8, CCL8, STAT1

To determine whether these functional interpretations remained stable under more stringent statistical criteria, enrichment analyses were repeated using the 52-gene *FDR*-adjusted core. Despite the reduction in gene-set size, the stringent core retained the principal biological signals identified using the expanded set, including antiviral defense, type I and type II interferon signaling, double-stranded RNA-associated responses, and cytokine and chemokine signaling. The multi-database enrichment results derived from the 52-gene core are presented in **Supplementary Figure 2**.

To identify central molecular interactions within this shared program, the expanded 164-gene set was mapped to the STRING database to construct a protein-protein interaction (PPI) network. The resulting network contained 160 nodes and 924 edges, with a highly significant enrichment *P* value < 0.001 (**Figure 2B**). Functional module analysis using the MCODE algorithm identified three major subnetworks within the PPI network (**Figure 2C**). Hub-gene analysis using the CytoHubba plugin further identified the top 20 hub genes, with STAT1, ISG15, and CXCL10 emerging as central nodes within the shared inflammatory network. Detailed information on all top 20 hub genes is provided in **Supplementary Table 3**. A subnetwork illustrating the interactions among these hub genes is shown in **Figure 2D**. A separate PPI analysis based on the stringent 52-gene core similarly retained STAT1, ISG15, and CXCL10 as prominent components of the shared interaction network, supporting the stability of the principal molecular findings after *FDR* correction (**Supplementary Figure 2B**). Complete differential-expression statistics for the hub genes in both datasets, including log_2_ fold changes, nominal *P* values, and Benjamini-Hochberg-adjusted *P* values, are provided in **Supplementary Table 4**. Collectively, the analyses of both the expanded 164-gene set and the stringent 52-gene core indicate convergence on a STAT1-centered, interferon-dominant host inflammatory program shared between SARS-CoV-2-associated hepatobiliary injury and BA.

To further explore the regulatory context of the shared DEGs, TF-gene and miRNA-gene interaction networks were constructed using NetworkAnalyst (**Supplementary Figures 3**, **4**). These analyses suggested that the shared transcriptional program may be regulated by multiple upstream TFs and miRNAs. Gene-disease association analysis further linked the shared DEGs to broader immune- and inflammation-related disease networks (**Supplementary Figure 5**), providing supplementary support for the biological relevance of the identified shared program.

### 3.3 Single-nucleus analyses localize the shared signature to cholangiocytes and indicate enrichment in states positioned earlier along the inferred trajectory

To define the cellular localization of the shared transcriptional program, we analyzed single-nucleus RNA-sequencing data from liver tissues with COVID-19-associated injury. Quality-control metrics and cell-type annotations are shown in **Supplementary Figure 6**, and the overall cellular landscape is presented in **Figure 3A**. Signature scores based on the expanded 164-gene shared set were first calculated at the single-nucleus level and subsequently averaged for each cell type within each donor. Donor-level comparisons showed that cholangiocytes exhibited among the highest mean signature scores across major hepatic cell populations (**Figure 3B**). In a paired within-donor analysis, the mean signature score was significantly higher in cholangiocytes than in all other hepatic cells combined (two-sided paired *Wilcoxon* signed-rank test, *P* = 0.00368), supporting preferential localization of the shared transcriptional program to the biliary epithelial compartment. This dataset lacked non-COVID-19 liver controls. We therefore used it only to assess the relative enrichment of the signature across cell types in COVID-19-associated liver injury, not to infer SARS-CoV-2-induced activation.

**Figure 3:**
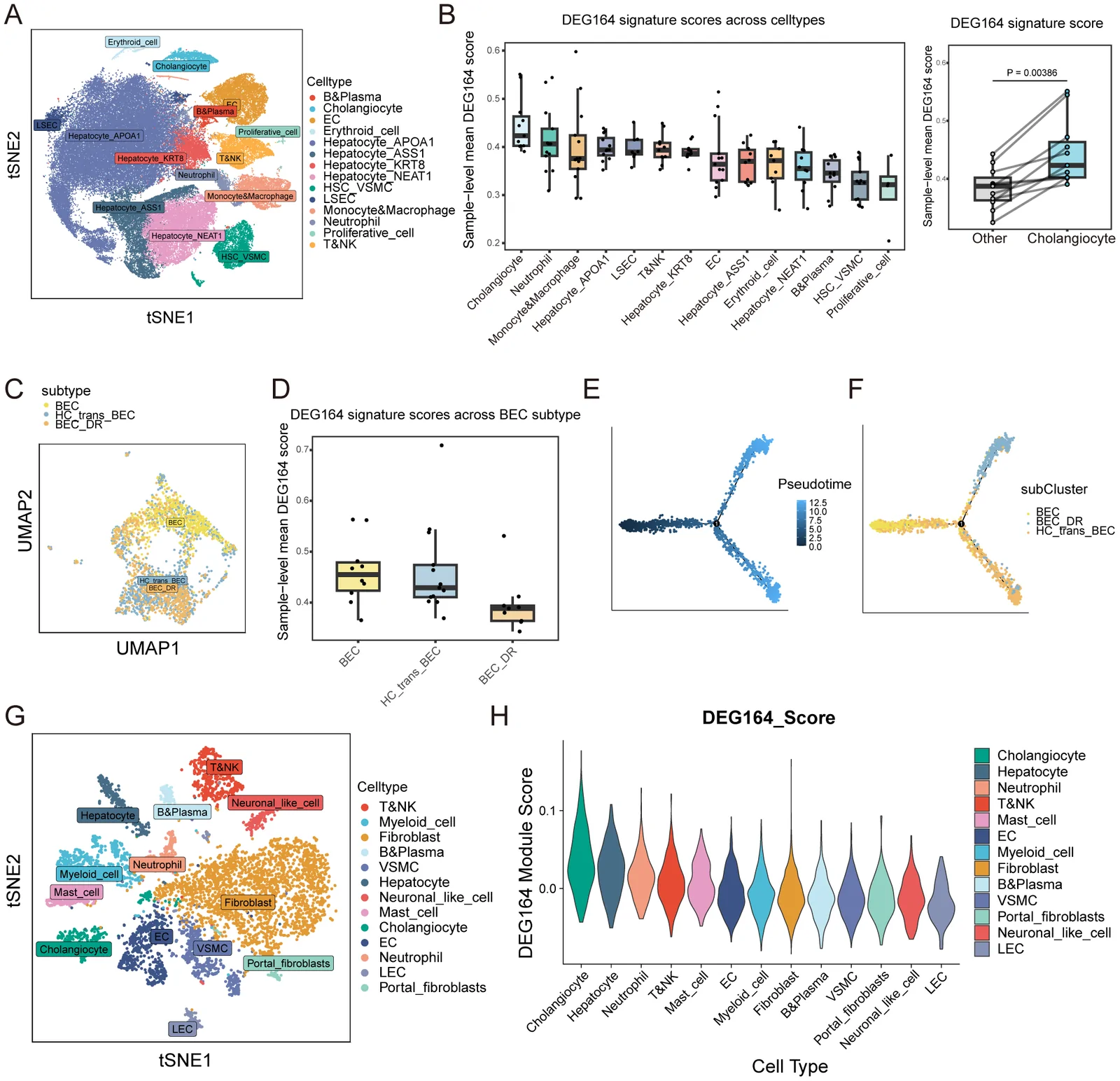
Single-nucleus analyses localize the expanded 164-gene shared signature to cholangiocytes. **(A)** *t*-SNE plot showing the major cell clusters from COVID-19 liver snRNA-seq colored by cell type. **(B)** Sample-level mean scores of the expanded 164-gene signature across major hepatic cell types. Signature scores were first calculated at the single-nucleus level and subsequently averaged within each cell type for each donor. Each point represents one independent donor. The paired plot compares the mean signature score of cholangiocytes with that of all other hepatic cells within the same donor; paired values from the same donor are connected by lines. Statistical significance was assessed using a two-sided paired *Wilcoxon* signed-rank test. **(C)** UMAP plot showing three distinct biliary epithelial cell (BEC) subpopulations, including BEC, HC_trans_BEC, and BEC_DR. **(D)** Sample-level mean scores of the expanded 164-gene signature across the three BEC subtypes. Signature scores were averaged for each BEC subtype within each donor, and each point represents one donor-subtype combination. **(E, F)** Pseudotime trajectory of BEC lineage inferred using the Monocle2. Cells are colored according to pseudotime and subtype, revealing lineage relationships among BEC, BEC_DR and HC_trans_BEC. **(G)** *t*-SNE plot of cell populations from BA snRNA-seq dataset. **(H)** 164-gene signature scores across major cell types in BA snRNA-seq dataset.

To further characterize cholangiocyte heterogeneity, cholangiocytes were extracted for subpopulation analysis and classified into three subsets: conventional cholangiocytes, reactive cholangiocytes, and hepatocyte-derived cholangiocytes (**Figure 3C**). Signature scores were first calculated at the single-nucleus level and subsequently averaged for each BEC subtype within each donor. At the donor level, conventional cholangiocytes showed the highest median score of the expanded 164-gene signature, whereas lower scores were observed in reactive and hepatocyte-derived cholangiocytes (**Figure 3D**). Pseudotime analysis placed the three BEC subtypes at partially distinct positions along an inferred branching trajectory, with conventional cholangiocytes more frequently distributed toward earlier regions and reactive or hepatocyte-derived cholangiocytes extending toward later branches (**Figures 3E, F**). The shared transcriptional program was more prominent in cholangiocytes at earlier pseudotime states. However, pseudotime does not establish the actual direction of cell differentiation.

To determine whether a similar pattern was present in BA, we performed single-nucleus RNA sequencing on liver tissue from one patient with BA. Quality control results are shown in **Supplementary Figures 7A–C**, and the corresponding cell clustering is presented in **Figure 3G**. Although trajectory analysis was not feasible in this single-sample dataset, cholangiocytes again showed the highest enrichment scores for the 164-gene signature (**Figure 3H**). Together, these findings identify cholangiocytes as the major cellular compartment associated with the shared transcriptional program in both SARS-CoV-2 associated hepatobiliary injury and BA.

### 3.4 Independent single-cell analysis validated cholangiocyte-associated activation of the shared signature in BA

To further validate the cholangiocyte-associated distribution of the shared transcriptional program, we analyzed an independent single-cell transcriptomic dataset including BA (n = 9) and choledochal cyst control (CC, n = 3) samples (**Supplementary Figures 7D, E**). After cell annotation, the major hepatic cell populations were visualized by *t*-SNE (**Figure 4A**). We then calculated the 164-gene signature score across different cell types. In this independent dataset, the shared signature was enriched in several cell populations, with particularly high scores observed in liver sinusoidal endothelial cells and cholangiocytes (**Figure 4B**). The shared signature was enriched in cholangiocytes in both the COVID-19 liver and our in-house BA single-nucleus datasets. We therefore focused subsequent cross-disease analyses on cholangiocytes, the principal epithelial cell type affected in BA and a vulnerable cell population in SARS-CoV-2-associated hepatobiliary injury. The 164-gene signature score was increased in BA cholangiocytes relative to CC cholangiocytes (**Figure 4C**). We next examined the expression of the three key genes highlighted by the upstream analyses. STAT1, ISG15, and CXCL10 were all upregulated in BA cholangiocytes compared with CC cholangiocytes (**Figures 4D–F**). These findings provide independent single-cell support for disease-associated activation of the shared cholangiocyte-associated host inflammatory program in BA and further substantiate the involvement of STAT1, ISG15, and CXCL10 in this cholangiocyte-associated inflammatory signature.

**Figure 4:**
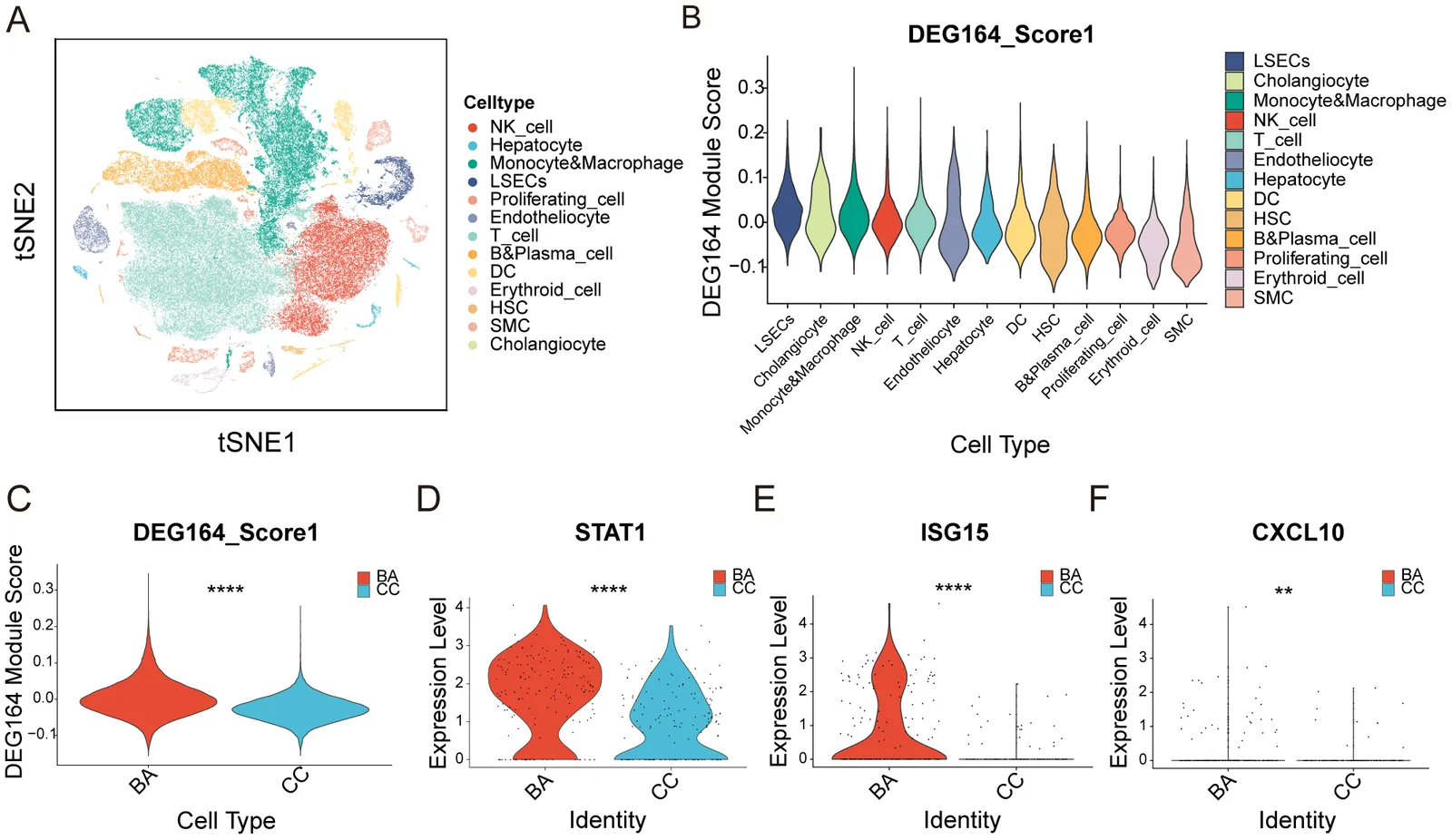
Independent single-cell validation of the shared cholangiocyte-associated transcriptional program in biliary atresia. **(A)** *t*-SNE visualization of the independent single-cell transcriptomic dataset from BA and CC liver tissues, showing the annotated major cell populations. **(B)** Distribution of the 164-gene shared signature score across major cell types. **(C)** Comparison of the 164-gene signature score in cholangiocytes between BA and CC samples. **(D–F)** Violin plots showing the expression of STAT1, ISG15, and CXCL10 in cholangiocytes from BA and CC samples. **P < 0.01; ****P < 0.0001.

### 3.5 Tissue and cellular analyses support a cholangiocyte-associated inflammatory program in BA

To provide orthogonal tissue-level corroboration of the cholangiocyte-associated inflammatory program identified by transcriptomic analyses, we examined the spatial expression of key markers in liver tissues from three patients with BA and one choledochal cyst (CC) control by immunofluorescence. Representative images are shown in **Figure 5A**, and three randomly selected, non-overlapping fields from each available specimen are provided in **Supplementary Figure 8**. Across the three BA specimens, pSTAT1 and total STAT1 signals appeared more prominent within KRT19-positive ductular structures than in the control tissue, with evident nuclear localization in cholangiocytes. ISG15 staining was also more intense in KRT19-positive cholangiocytes in the BA specimens. CXCL10 exhibited a predominantly periductal distribution, with stronger staining surrounding ductular structures in BA tissues, consistent with its role as a chemokine within the local inflammatory microenvironment. Given the limited number of technically suitable control specimens, these findings are interpreted as exploratory spatial and protein-level corroboration rather than definitive cohort-level validation.

**Figure 5:**
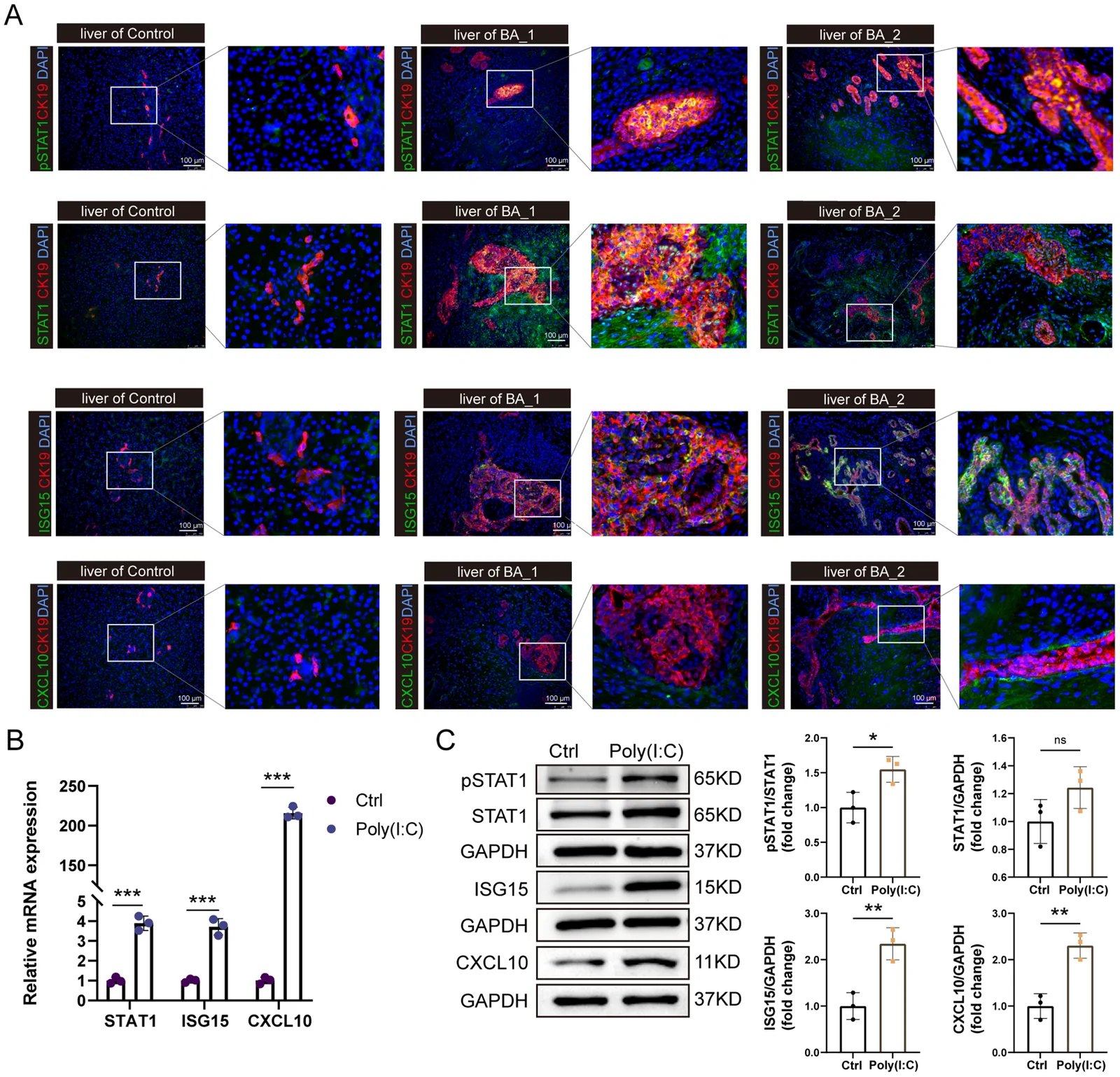
Experimental support of the activation of cholangiocyte-associated inflammatory axis in BA. **(A)** Immunofluorescence staining of control and BA livers, ×200. Green staining indicates pSTAT1, STAT1, ISG15 and CXCL10. Red staining indicates KRT19 and blue staining (DAPI) indicates the cell nucleus. **(B)** Relative mRNA expression levels of STAT1, ISG15 and CXCL10 in H69 cholangiocytes treated with poly(I:C) (30 μg/mL) for 24h, as determined by qRT-PCR. **(C)** The proteins expression level in human cholangiocytes, cells were treated with poly(I:C) 30 μg/mL for 24h. Data are presented as mean ± SD from three independent biological experiments, *t* test; *P < 0.05; **P < 0.01; ***P < 0.001; ns, not significant.

To determine whether cholangiocytes are intrinsically capable of activating representative components of the shared interferon/inflammatory program in response to a viral-mimetic stimulus, H69 cholangiocytes were treated with poly(I:C), a synthetic double-stranded RNA mimic, at 30 μg/mL for 24 h. qRT-PCR analysis showed that STAT1, ISG15, and CXCL10 mRNA levels were significantly increased following poly(I:C) stimulation, with CXCL10 exhibiting the most pronounced induction (**Figure 5B**). At the protein level, poly(I:C) significantly increased the pSTAT1/STAT1 ratio, indicating enhanced STAT1 phosphorylation, whereas total STAT1 protein showed a nonsignificant upward trend. ISG15 and CXCL10 protein levels were also significantly increased in poly(I:C)-treated cells (**Figure 5C**). These findings show that cholangiocytes are intrinsically capable of activating representative components of the identified STAT1-associated interferon/inflammatory response following double-stranded RNA-related stimulation. Together with the epithelial organoid and single-cell localization findings, these results provide complementary experimental support for the cholangiocyte-associated transcriptional program identified in SARS-CoV-2-associated hepatobiliary injury and BA.

### 3.6 Computational drug prioritization and molecular docking support mefloquine as an exploratory candidate

To explore the potential therapeutic implications of the shared transcriptional program, drug-gene interaction analysis was initially performed using Enrichr based on the DSigDB database and the expanded 164-gene exploratory shared set. According to the enrichment *P* values, the top 10 candidate compounds were prenylamine, acetohexamide, suloctidil, chlorophyllin, terfenadine, rolipram, cortisol succinate, prochlorperazine, mefloquine, and triprolidine (**Table 4**). A heatmap illustrating the associations between the top-ranked compounds and representative shared genes is shown in **Figure 6A**. STAT1, ISG15, and CXCL10 were recurrently represented across the predicted drug-gene interaction network.

**Table 4:** Candidate drugs (top 10) identified from gene–drug interaction enrichment analysis.

Name	P-value	Chemical formula	Structure
prenylamine HL60 UP	2.88E-20	C_24_H_27_N	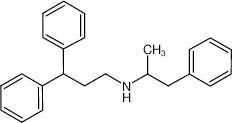
acetohexamide PC3 UP	1.09E-15	C_15_H_20_N_2_O_4_S	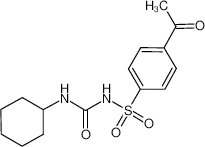
suloctidil HL60 UP	5.15E-25	C_20_H_35_NOS	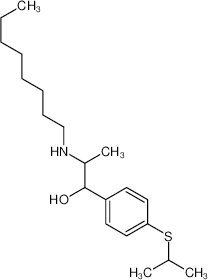
chlorophyllin CTD 00000324	3.81E-09	C_34_H_36_N_4_O_6_
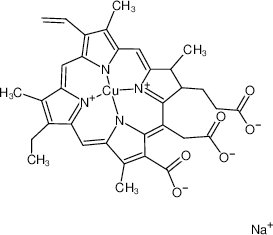
terfenadine HL60 UP	5.52E-11	C_32_H_41_NO_2_	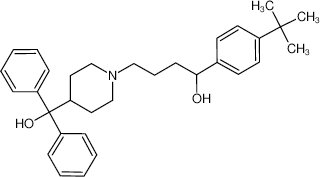
rolipram CTD 00007371	5.54E-06	C_16_H_21_NO_3_	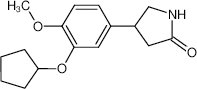
cortisol succinate CTD 000000336	8.51E-05	C_25_H_34_O_8_	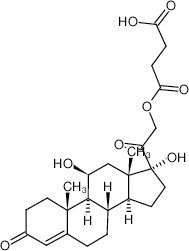
prochlorperazine MCF7 UP	2.33E-08	C_20_H_24_ClN_3_S	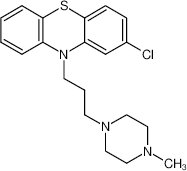
mefloquine HL60 UP	3.53E-05	C_17_H_16_F_6_N_2_O	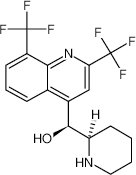
triprolidine PC3 UP	2.84E-05	C_19_H_22_N_2_	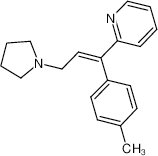

**Figure 6:**
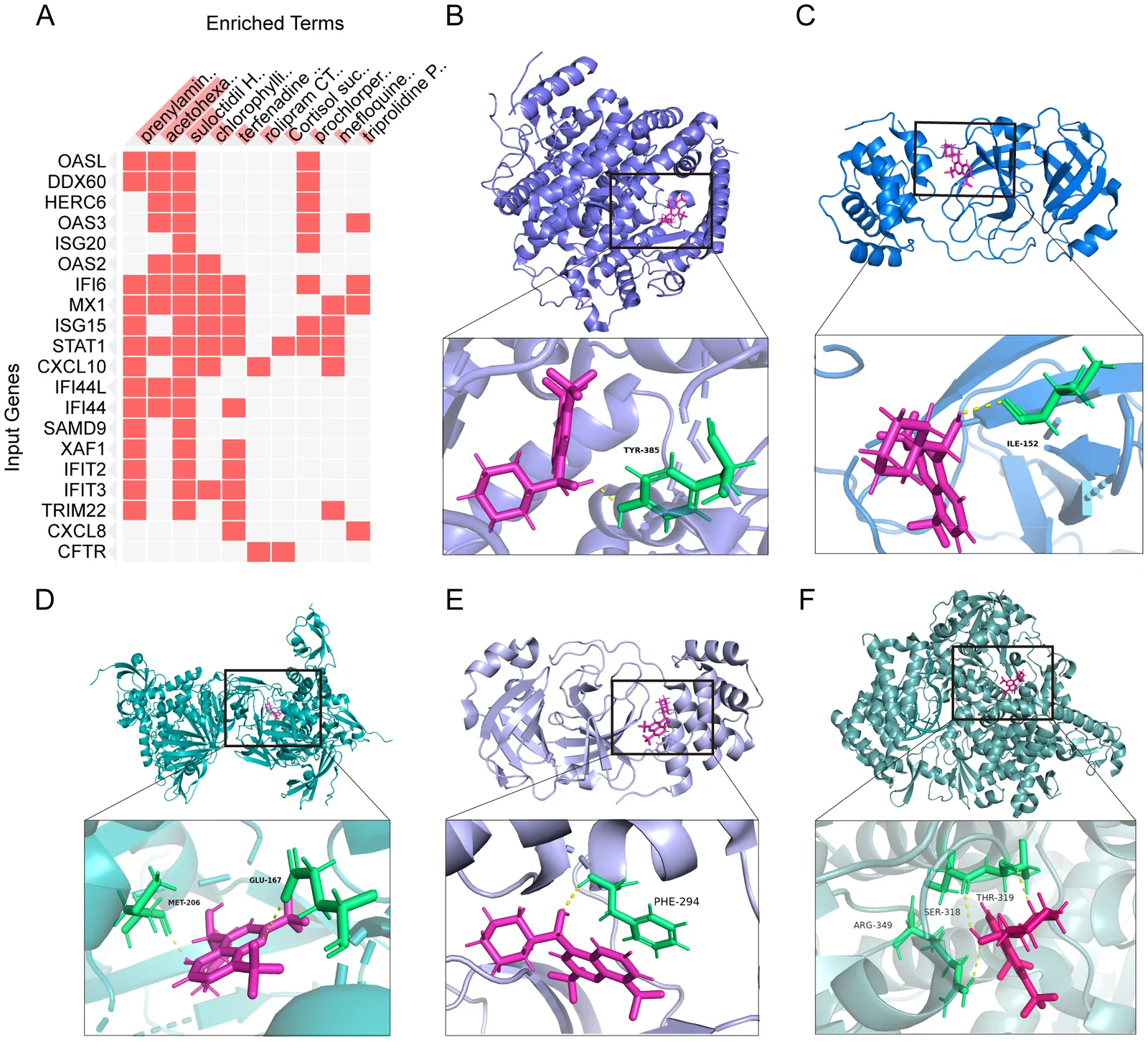
Candidate drug prioritization and representative molecular docking analysis. **(A)** Heatmap showing the associations between the top predicted candidate compounds and key shared genes, highlighting the contributions of STAT1, ISG15 and CXCL10 to the predicted drug-gene interaction network. **(B–F)** Representative molecular docking models of mefloquine with five SARS-CoV-2-related targets: ACE2, 3CLpro, Mpro, PLpro, and RdRp, respectively.

To evaluate the robustness of the drug-prioritization results under a more stringent gene-selection strategy, the DSigDB analysis was repeated using the 52-gene *FDR*-adjusted shared core. Mefloquine remained among the top 10 predicted compounds, indicating that its computational prioritization was retained after multiple-testing correction. The drug-gene association pattern derived from the stringent 52-gene core is shown in **Supplementary Figure 9M**, and the corresponding enrichment results are provided in **Supplementary Table 5**.

Molecular docking was subsequently performed to explore the predicted interactions of the prioritized compounds with five SARS-CoV-2-related targets, including ACE2, 3CLpro, Mpro, PLpro, and RdRp. Most compound-target pairs yielded AutoDock Vina scores below −5.0 kcal/mol under the applied docking conditions (**Table 5**). Among the prioritized compounds, mefloquine showed consistently favorable predicted docking scores across multiple SARS-CoV-2-related targets and was therefore selected for representative structural visualization in **Figures 6B–F**. Docking results for the other top-ranked compounds are provided in **Supplementary Figures 9A–J**.

**Table 5:** Binding energies of candidate compounds against SARS-CoV-2-related targets as evaluated by AutoDock Vina (kcal/mol).

Name	Chemical formula	ACE2	3CLpro	Mpro	PLpro	RdRp
prenylamine	C_24_H_27_N	-8.3	-6.4	-7.7	-6.8	-6.4
acetohexamide	C_15_H_20_N_2_O_4_S	-7.9	-7.0	-7.7	-7.0	-6.7
suloctidil	C_20_H_35_NOS	-6.3	-4.6	-5.7	-5.3	-5.4
chlorophyllin	C_34_H_36_N_4_O_6_	-7.8	-7.2	-8.9	-6.8	-6.9
terfenadine	C_32_H_41_NO_2_	-8.5	-6.5	-8.6	-7.9	-6.7
rolipram	C_16_H_21_NO_3_	-6.7	-6.0	-7.5	-6.4	-6.4
cortisol succinate	C_25_H_34_O_8_	-7.5	-7.4	-8.7	-6.8	-6.8
prochlorperazine	C_20_H_24_ClN_3_S	-7.1	-6	-8.6	-6.7	-7.3
mefloquine	C_17_H_16_F_6_N_2_O	-8.0	-7.2	-9.3	-7.7	-7.3
triprolidine	C_19_H_22_N_2_	-7.2	-6.3	-7.4	-6.4	-6.8

Because the original docking assessment primarily focused on SARS-CoV-2-related proteins, we further extended the exploratory structural analysis to viral proteins selected from BA-relevant clinical and experimental contexts. Mefloquine yielded a top-ranked docking score of −7.2 kcal/mol against the HCMV UL80 protease and −6.4 kcal/mol against a structurally characterized rotavirus VP1 RNA-dependent RNA polymerase. Representative docking conformations are shown in **Supplementary Figures 9K, L**, and the corresponding docking results are summarized in **Supplementary Table 6**.

Collectively, the reproducibility of mefloquine prioritization using both the expanded 164-gene set and the stringent 52-gene core, together with its predicted docking interactions across SARS-CoV-2-related and BA-relevant viral contexts, supports its selection as an exploratory candidate for future investigation. However, these analyses remain hypothesis-generating and do not establish direct target binding, enzymatic inhibition, antiviral efficacy, or therapeutic activity in BA.

## 4 Discussion

Biliary atresia (BA) is a severe neonatal cholangiopathy characterized by progressive inflammation and fibrosis of the bile ducts that can ultimately progress to end-stage liver disease (22, 23). Although Kasai portoenterostomy can restore bile flow in some patients, many children still develop progressive hepatic dysfunction and eventually require liver transplantation (24, 25). Viral exposure has long been proposed as one of the potential triggers of BA, but the mechanisms by which infectious or inflammatory insults contribute to bile-duct-specific injury remain incompletely understood (26, 27). In parallel, hepatobiliary abnormalities have been increasingly recognized in patients with COVID-19, suggesting that the biliary epithelium may be involved in SARS-CoV-2-associated extrapulmonary injury (28, 29). In this study, we integrated transcriptomic data from SARS-CoV-2-infected human liver organoids and BA liver tissues with single-nucleus, single-cell, tissue-level, and cell-based evidence to examine whether these biologically distinct injury contexts converge on a cholangiocyte-associated inflammatory host-response program. Our analyses aim to identify molecular convergence, rather than establishing a common etiology or causal relationship between SARS-CoV-2-associated hepatobiliary injury and BA.

The initial cross-disease analysis identified the expanded 164-gene shared set between SARS-CoV-2-associated hepatobiliary injury and BA. Because this expanded gene set was defined using a nominal significance threshold, it was interpreted as exploratory. We therefore repeated the analysis using a stringent threshold of |log_2_ fold change| > 1 and Benjamini-Hochberg-adjusted *P* < 0.05, which identified a 52-gene *FDR*-adjusted core. The 52-gene core was entirely contained within the expanded set and retained STAT1, ISG15, and CXCL10. Both gene sets consistently implicated interferon signaling, antiviral defense, double-stranded RNA-associated responses, and cytokine and chemokine signaling, supporting the robustness of the principal biological interpretation across different statistical thresholds. These findings suggest that the two conditions share a measurable host-response signature at the transcriptomic level. Importantly, this overlap should not be interpreted as evidence that SARS-CoV-2 infection causes BA or that the two diseases are mechanistically identical. STAT1, ISG15, and CXCL10 are canonical interferon-responsive genes that can be induced in multiple inflammatory conditions and therefore cannot be considered specific to BA or cholangiocyte injury. Nevertheless, the detection of this program in liver ductal organoids, which are predominantly epithelial and lack the complex immune-cell compartment of whole liver tissue, indicates that the observed signal cannot be attributed solely to immune-cell infiltration. The findings instead support convergence of distinct injury contexts on a recurrent epithelial interferon/inflammatory response.

Network analysis further highlighted STAT1, ISG15, and CXCL10 as central components of this shared program. These genes may represent complementary components of a cholangiocyte-associated inflammatory response rather than disease-specific biomarkers or proven causal mediators. STAT1 is a key mediator of type I and type II IFN signaling and regulates a broad set of IFN-stimulated genes involved in antiviral defense, antigen presentation, and inflammatory amplification (30–33). Sustained STAT1 signaling within cholangiocytes may therefore maintain an epithelial antiviral and inflammatory state, although the present study does not establish that STAT1 activation directly causes bile duct obstruction or fibrosis. ISG15 is a canonical interferon-stimulated effector involved in intracellular antiviral defense and protein ISGylation (34, 35). Its increased representation in cholangiocytes is consistent with activation of an epithelial innate immune-response state, but a direct injurious role for ISG15 was not examined. CXCL10 is an interferon-responsive chemokine downstream of STAT1 that may promote the recruitment of CXCR3-expressing immune cells to the periductal compartment (36). Previous studies have also implicated CXCL10 in BA-associated biliary inflammation (37, 38). Its predominantly periductal distribution in BA tissues in the present study therefore suggests a potential link between epithelial activation and the local inflammatory microenvironment. Coordinated expression of STAT1, ISG15, and CXCL10 may therefore delineate a putative epithelial-to-immune inflammatory axis that amplifies biliary inflammation; however, its causal contribution to BA pathogenesis requires direct functional testing.

A major strength of the study is the cellular resolution provided by the single-nucleus analyses. In the COVID-19 liver single-nucleus dataset, donor-level analysis demonstrated preferential localization of the shared signature to cholangiocytes, whose mean signature scores were significantly higher than those of all other hepatic cells combined. Because this dataset did not contain non-COVID-19 controls, the result should be interpreted as relative cellular localization within COVID-19-associated liver injury rather than evidence of disease-induced activation relative to normal liver. Further subclustering showed that the signal was most evident in conventional cholangiocytes, and inferred trajectory analysis suggested enrichment in cholangiocyte states positioned earlier along the trajectory. Although this does not establish a true temporal sequence, it raises the possibility that activation of the shared interferon-related program is associated with relatively early phases of cholangiocyte injury. This point is relevant because early molecular alterations in BA are difficult to capture clinically. BA is usually recognized after obstructive jaundice becomes apparent, whereas the initial epithelial stress responses preceding overt bile duct obstruction remain poorly defined (39, 40). Our findings therefore provide a transcriptomic clue that interferon-related signaling may be engaged early in cholangiocyte injury. This interpretation, however, should remain cautious, since the early-state inference was derived from the COVID-19 single-nucleus dataset rather than from longitudinal BA samples.

The BA single-nucleus dataset generated in this study provided an additional layer of support. Because only one BA specimen was available, this dataset was used for descriptive cellular localization and not for patient-level statistical inference or independent validation. Although the shared signature was also enriched in other hepatic cell populations, including liver sinusoidal endothelial cells, the in-house dataset showed prominent representation of the signature within the cholangiocyte compartment. An independent BA single-cell cohort further supported increased signature scores and greater representation of STAT1-, ISG15-, and CXCL10-associated signals in BA cholangiocytes relative to controls. These observations reduce the likelihood that the bulk-tissue signal arose exclusively from variation in immune-cell abundance. Nevertheless, the program was not restricted to cholangiocytes, and the available data do not establish cholangiocyte or disease specificity.

Tissue and cell-based experiments provided additional, but still exploratory, support for the transcriptomic observations. In BA liver tissues, pSTAT1/STAT1 and ISG15 signals were spatially associated with KRT19-positive ductular epithelial structures, whereas CXCL10 was predominantly detected in the periductal compartment. These findings link representative components of the program to regions of biliary epithelial and periductal injury. However, because the tissue cohort included three BA specimens and one technically suitable control, the immunofluorescence results should be regarded as exploratory spatial corroboration rather than definitive cohort-level validation. To examine whether cholangiocytes themselves are capable of mounting the identified response, cultured human cholangiocytes were exposed to poly(I:C), a synthetic double-stranded RNA mimic. Poly(I:C) induced representative components of the shared interferon/inflammatory program, including STAT1, ISG15, and CXCL10. Together with the epithelial organoid findings, these results support the intrinsic capacity of cholangiocytes to respond to viral nucleic acid-associated molecular stimuli and argue against the possibility that the observed transcriptional signal is attributable solely to infiltrating immune cells. Nevertheless, poly(I:C) exposure represents a reductionist viral-mimetic model and does not reproduce authentic viral infection, viral replication, or the complete host response to SARS-CoV-2. These experiments also do not establish viral infection as the upstream cause of the inflammatory program observed in human BA.

Several viruses, including rotavirus and cytomegalovirus, have previously been implicated in BA through immune-mediated bile duct injury (26, 41–43). However, viral associations have not been consistently demonstrated across all BA cohorts, and BA remains an etiologically heterogeneous disorder. The present study therefore does not identify SARS-CoV-2 as a cause of BA or imply that all BA cases arise from viral exposure. Rather, it supports a broader model in which distinct infectious or inflammatory contexts may converge on overlapping downstream host-response programs within a vulnerable biliary epithelial compartment. In this framework, SARS-CoV-2-associated hepatobiliary injury serves as a comparative context for identifying epithelial inflammatory responses potentially relevant to BA, rather than as an etiological model of BA itself.

The computational drug-prioritization analysis should nevertheless be interpreted cautiously. Mefloquine was initially identified among the top-ranked compounds using the expanded 164-gene exploratory set and remained among the top 10 candidates when the analysis was repeated using the stringent 52-gene *FDR*-adjusted core. This concordance supports the internal stability of the computational ranking but does not establish target engagement, biological activity, or therapeutic relevance. Molecular docking further showed favorable predicted docking compatibility between mefloquine and multiple SARS-CoV-2-related proteins. To address the initially limited connection between the SARS-CoV-2-focused docking analysis and the BA component of the study, the structure-based assessment was extended to the HCMV UL80 protease and a structurally characterized rotavirus VP1 RNA-dependent RNA polymerase, representing viral contexts previously implicated in clinical BA research (44, 45). These additional analyses broadened the hypothesis-generating computational assessment but did not demonstrate that mefloquine acts on BA-associated inflammatory pathways. AutoDock Vina scores represent predicted docking poses and relative compatibility rather than experimentally measured binding affinity or binding free energy. The docking results therefore do not establish direct target engagement, enzymatic inhibition, antiviral activity, or therapeutic efficacy in BA.

The computational prioritization of mefloquine is consistent with previous *in vitro* reports of activity against SARS-CoV-2, but these external findings should not be considered validation of the present predictions. Shionoya et al. demonstrated that mefloquine inhibited SARS-CoV-2 infection in both VeroE6/TMPRSS2 and human Calu-3 cells, with low-micromolar inhibitory concentrations, and identified inhibition of viral entry after attachment as a principal mechanism (46). Gendrot et al. independently reported dose-dependent inhibition of SARS-CoV-2 replication by mefloquine in Vero E6 cells (47). In addition, Sacramento et al. showed that mefloquine suppressed SARS-CoV-2 replication in Calu-3 cells and monocytes, impaired viral entry, and attenuated virus-associated induction of IL-6 and TNF-α in monocytes (48). These observations support only the broader biological plausibility of mefloquine as an antiviral compound. They do not validate the specific docking poses generated here, demonstrate activity in cholangiocytes, or show modulation of STAT1, ISG15, CXCL10, or biliary injury. Direct biochemical target-engagement studies, cholangiocyte intervention experiments, dose-response analyses, and *in vivo* studies are required before any therapeutic implication can be considered.

This study has several limitations. First, the initial cross-disease comparison integrated SARS-CoV-2-infected liver organoids with BA liver tissues. These systems differ substantially in donor age, cellular composition, tissue architecture, immune-cell content, experimental context, and injury stage, precluding direct biological equivalence. Although the COVID-19 liver single-nucleus dataset provided cell-type-resolved support, its lack of non-COVID-19 controls allowed assessment of preferential cellular localization but not disease-associated induction relative to normal liver. Second, the in-house BA snRNA-seq dataset comprised a single specimen and was therefore used only for descriptive cellular localization rather than donor-level inference. The number of control donors in the independent BA single-cell cohort was also limited. Similarly, the tissue immunofluorescence cohort included three BA specimens and one technically suitable control, and multiple fields from the same specimen represented spatial sampling rather than independent biological replicates. These findings therefore require validation in larger, independently collected and clinically annotated cohorts using sample-aware analytical approaches. Third, pseudotime analysis was inferred from cross-sectional data and cannot establish a true temporal sequence or lineage transition. Poly(I:C) stimulation demonstrated the inducibility of representative genes in cholangiocytes but did not reproduce authentic viral infection or establish a viral cause of BA. Because other inflammatory or fibrosing cholangiopathies were not included as disease controls, the specificity of the identified program relative to other cholangiopathies remains undetermined. Finally, the present study is primarily associative. It does not establish that STAT1, ISG15, or CXCL10 causally drive biliary injury, nor do the computational drug-prioritization and docking analyses demonstrate target engagement or therapeutic efficacy. These hypotheses require direct genetic, pharmacological, biochemical, and *in vivo* validation.

In summary, this study supports a convergent cholangiocyte-associated interferon/inflammatory host-response program in SARS-CoV-2-associated hepatobiliary injury and BA. The combined bulk transcriptomic, donor-level single-nucleus, independent single-cell, tissue-level, and cholangiocyte stimulation data identify STAT1, ISG15, and CXCL10 as representative components of this program. The findings demonstrate molecular association and cellular localization rather than a common etiology, disease specificity, or causal mechanism. They provide a systems-level framework and testable hypotheses for further investigation of biliary epithelial inflammatory responses, while the diagnostic and therapeutic relevance of the identified genes and computationally prioritized compounds remains to be established.

## Data Availability

The datasets presented in this study can be found in online repositories. The names of the repository/repositories and accession number(s) can be found below: https://ngdc.cncb.ac.cn/omix, OMIX018192.
