# Supplementary material for: Integrative transcriptomic and single-cell analysis reveals a convergent cholangiocyte-associated host inflammatory program in biliary atresia and SARS-CoV-2-associated hepatobiliary injury
Source: Front Immunol. 2026 Sep 16;17:1923630. doi: 10.3389/fimmu.2026.1923630 (PMC13624073; doi:10.3389/fimmu.2026.1923630)
Supplement: Supplementary file 1 [file SupplementaryFile1.docx]

Supplementary Materials

# 1 Supplementary Figures

# Supplementary Figure S1

**Figure S1. The bar graphs of pathway enrichment analysis of the expanded 164-gene shared set between SARS-CoV-2 and biliary atresia performed by the Enrichr.** (A) Reactome, (B) BioPlanet, (C) WikiPathway and (D) KEGG.

# Supplementary Figure S2

**Figure S2. Functional characterization of the stringent *FDR*-adjusted shared gene set in SARS-CoV-2 and biliary atresia.** (A) Venn diagram showing the overlap between differentially expressed genes identified in the SARS-CoV-2-infected liver organoid dataset GSE210058 and the biliary atresia liver dataset GSE159720 using the revised multiple-testing-adjusted significance criteria. A total of 52 genes were shared between the two datasets. (B) Protein–protein interaction network of the 52 shared genes, demonstrating a highly interconnected antiviral and interferon-related module containing STAT1, ISG15, CXCL10, MX1, OAS family members, and other interferon-stimulated genes. (C-H) Functional enrichment analysis of the stringent 52-gene shared set using Gene Ontology Biological Process (C), Gene Ontology Molecular Function (D), Reactome (E), BioPlanet (F), WikiPathways (G), and KEGG databases (H).

**Supplementary Figure S3**

**Figure S3.** **The DEGs-TFs regulatory interaction network.** The Network Analyst provided the DEG-TFs regulatory interaction network. TFs are represented by blue diamonds, while common DEGs are represented by red circles. The size of circles and diamonds represent the degree of nodes.

**Supplementary Figure S4**

**Figure. S4.** **DEGs-miRNAs regulatory interaction network.** The DEGs-miRNAs regulation interaction network. Green circles represent DEGs, and the purple squares represent miRNAs.

**Supplementary Figure S5**

**Figure S5. Common DEGs-associated disorders are represented by the gene-disease association network.** The red circular nodes defined their gene symbols, whereas the blue square nodes indicated the disorders.

**Supplementary Figure S6**

**Figure S6. Quality control of snRNA-seq dataset from COVID-19.** (A) Violin plots showing quality control across samples, including the nFeature_RNA, nCount_RNA, percent.mt. (B) *t*-SNE plot of all nuclei after filtering and batch correction. Each color represents a distinct cluster. (C) Stacked bar plot showing the proportions of each annotated cell type across covid-19 liver samples. (D) Dot plot displays marker genes expression in cell clusters for cell type annotation. (E) Violin plots showing the expression of BEC markers, ductular reaction markers and hepatocyte markers.

**Supplementary Figure S7**

**Figure S7.** **Quality control and cell-type annotation of the in-house BA snRNA-seq dataset and the independent public scRNA-seq dataset (GSE176189).** (A) Violin plots showing quality-control metrics of singlet nuclei from the BA liver snRNA-seq dataset generated in this study after doublet removal, including nFeature_RNA, nCount_RNA, and percent.mt. (B) *t*-SNE plot of the filtered singlet nuclei from our BA snRNA-seq dataset used for downstream analyses. (C) Dot plot showing the expression of representative marker genes across annotated cell populations in our BA snRNA-seq dataset. (D) *t*-SNE plot of the independent public scRNA-seq dataset (GSE176189) used for external validation. (E) Dot plot showing the expression of representative marker genes across annotated cell populations in the independent public scRNA-seq dataset (GSE176189).

**Supplementary Figure S8**

**Figure S8. Immunofluorescence localization of STAT1-associated inflammatory markers in BA and CC liver.** Representative immunofluorescence images of phosphorylated STAT1 (pSTAT1) (A), total STAT1 (B), ISG15 (C), and CXCL10 (D) in liver tissues from one choledochal cyst control and three patients with biliary atresia.

**Supplementary Figure S9**

**Figure S9. Representative molecular docking models of other top-ranked candidate compounds with SARS-CoV-2 infection and BA related targets.** Representative docking models showing the interactions of ACE2 with terfenadine (A) and prenylamine (B); 3CLpro with cortisol succinate (C) and chlorophyllin (D); Mpro with chlorophyllin (E) and cortisol succinate (F); PLpro with terfenadine (G) and acetohexamide (H); and RdRp with prochlorperazine (I) and chlorophyllin (J). Representative top-ranked docking poses of mefloquine with (K) a structurally characterized rotavirus VP1 RNA-dependent RNA polymerase (PDB ID: 2R7X) and (L) the HCMV UL80 protease (PDB ID: 8J3T). (M) Drug-gene association heatmap based on the stringent 52-gene shared signature.

# 2. Supplementary Tables

**Supplementary Table 1. Primer sequence of qRT-PCR**

| **Primer name** | **Primer sequence** |
| --- | --- |
| GAPDH-F | 5’-AAGGGTCATCATCTCTGCCC-3’ |
| GAPDH-R | 5’-CATGGACTGTGGTCATGAGT-3’ |
| STAT1-F | 5’-TCTGTGTCTGAAGTTCACCCTT-3’ |
| STAT1-R | 5’-CCGAGACACCTCGTCAAACT-3’ |
| ISG15-F | 5’-CTCTGAGCATCCTGGTGAGGAA-3’ |
| ISG15-R | 5’-AAGGTCAGCCAGAACAGGTCGT-3’ |
| CXCL10-F | 5’-AGTGGCATTCAAGGAGTACCT-3’ |
| CXCL10-R | 5’-CGTGGACAAAATTGGCTTGC-3’ |

| **number** | **gene** | **number** | **gene** | **number** | **gene** | **number** | **gene** |
| --- | --- | --- | --- | --- | --- | --- | --- |
| 1 | ISG15 | 42 | STAP1 | 83 | LSP1 | 124 | ISG20 |
| 2 | ESPN | 43 | UGT2A3 | 84 | TRIM22 | 125 | ANPEP |
| 3 | AGMAT | 44 | SULT1E1 | 85 | LYVE1 | 126 | NR2F2-AS1 |
| 4 | KLHDC7A | 45 | CXCL8 | 86 | F2 | 127 | MMP25 |
| 5 | PLA2G2A | 46 | AREG | 87 | GLYATL1 | 128 | SLC5A11 |
| 6 | UBXN10 | 47 | CXCL10 | 88 | BATF2 | 129 | CES3 |
| 7 | IFI6 | 48 | CXCL11 | 89 | P2RY6 | 130 | HSD11B2 |
| 8 | TINAGL1 | 49 | HERC6 | 90 | ITIH5 | 131 | HYDIN |
| 9 | PCSK9 | 50 | DDX60 | 91 | CUBN | 132 | DPEP1 |
| 10 | IFI44L | 51 | DDX60L | 92 | RET | 133 | PITPNM3 |
| 11 | IFI44 | 52 | C6 | 93 | GDF2 | 134 | XAF1 |
| 12 | GBP1 | 53 | ANKDD1B | 94 | PHYHIPL | 135 | LINC00324 |
| 13 | GBP6 | 54 | PCDHGA8 | 95 | RTKN2 | 136 | DNAH9 |
| 14 | TGFBR3 | 55 | TIMD4 | 96 | HKDC1 | 137 | EFCAB5 |
| 15 | EPS8L3 | 56 | HAVCR1 | 97 | OIT3 | 138 | ASIC2 |
| 16 | FCGR1A | 57 | ITK | 98 | MMRN2 | 139 | CCL8 |
| 17 | S100A4 | 58 | FGFR4 | 99 | IFIT2 | 140 | SLFN13 |
| 18 | NPR1 | 59 | FOXQ1 | 100 | IFIT3 | 141 | HNF1B |
| 19 | TNN | 60 | DCDC2 | 101 | CYP26A1 | 142 | NGFR |
| 20 | IL10 | 61 | TRIM31 | 102 | FGF23 | 143 | CD300LF |
| 21 | CMPK2 | 62 | PSMB9 | 103 | PLEKHG6 | 144 | RNF213 |
| 22 | XDH | 63 | PTCHD4 | 104 | KLRD1 | 145 | LAMA1 |
| 23 | CAPG | 64 | LINC00472 | 105 | SLC39A5 | 146 | LAMA3 |
| 24 | NEURL3 | 65 | UNC93A | 106 | OAS3 | 147 | ONECUT2 |
| 25 | MYO7B | 66 | AGR2 | 107 | OAS2 | 148 | SEMA6B |
| 26 | THSD7B | 67 | DNAH11 | 108 | HNF1A | 149 | ANGPTL6 |
| 27 | FAP | 68 | LINC01176 | 109 | OASL | 150 | ACP5 |
| 28 | GALNT3 | 69 | SFRP4 | 110 | EPSTI1 | 151 | B3GNT3 |
| 29 | STAT1 | 70 | SEMA3E | 111 | TNFSF13B | 152 | FCGBP |
| 30 | AOX1 | 71 | SAMD9 | 112 | PLEKHH1 | 153 | CBLC |
| 31 | RUFY4 | 72 | SAMD9L | 113 | BATF | 154 | C5AR2 |
| 32 | VIL1 | 73 | MUC3A | 114 | TC2N | 155 | FUT2 |
| 33 | ACKR2 | 74 | CFTR | 115 | AMN | 156 | PLEKHA4 |
| 34 | CDCP1 | 75 | MAOB | 116 | ASPG | 157 | HRC |
| 35 | ZDHHC23 | 76 | GJB1 | 117 | C15orf62 | 158 | MYH14 |
| 36 | MUC13 | 77 | IDO1 | 118 | CYP19A1 | 159 | NLRP12 |
| 37 | SLCO2A1 | 78 | TTPA | 119 | ONECUT1 | 160 | CLDN5 |
| 38 | ARL14 | 79 | COLEC10 | 120 | WDR72 | 161 | ISX |
| 39 | LAMP3 | 80 | LY6E | 121 | LIPC | 162 | SOX10 |
| 40 | SLC34A2 | 81 | ANO9 | 122 | CA12 | 163 | SAMSN1 |
| 41 | RHOH | 82 | CDHR5 | 123 | PAQR5 | 164 | MX1 |

**Supplementary Table 2. The shared expanded 164-gene**

**Supplementary Table 3. Top 20 in network string_interactions_short.tsv ranked by MNC method**

| **Rank** | **Name** | **Score** |
| --- | --- | --- |
| 1 | STAT1 | 45 |
| 2 | ISG15 | 40 |
| 3 | CXCL10 | 36 |
| 4 | MX1 | 34 |
| 5 | EGFR | 32 |
| 5 | OASL | 32 |
| 7 | IRF1 | 30 |
| 8 | TP53 | 29 |
| 8 | PSMB9 | 29 |
| 8 | IL10 | 29 |
| 11 | IFI44 | 28 |
| 11 | IFIT3 | 28 |
| 11 | GBP1 | 28 |
| 14 | USP18 | 27 |
| 14 | IFIT2 | 27 |
| 14 | DDX60 | 27 |
| 17 | OAS3 | 26 |
| 17 | XAF1 | 26 |
| 17 | OAS2 | 26 |
| 17 | CXCL8 | 26 |

| **GSE210058** | **log_2_FC** | ***P* value** | **adjusted *P* value** |  | **GSE159720** | **log_2_FC** | ***P* value** | **adjusted**  ***P* value** |
| --- | --- | --- | --- | --- | --- | --- | --- | --- |
| ISG15 | 4.752998 | 1.17E-24 | 3.89E-21 |  | ISG15 | 2.192704 | 1.25E-05 | 0.000723684 |
| IFI44 | 4.216418 | 1.02E-19 | 1.3E-16 |  | IFI44 | 1.954967 | 0.000893 | 0.016206514 |
| STAT1 | 2.557007 | 1.1E-13 | 7.59E-11 |  | STAT1 | 1.300483 | 0.000366 | 0.008655508 |
| CXCL10 | 5.355044 | 1.08E-06 | 0.000152 |  | CXCL10 | 4.660587 | 1.38E-08 | 3.64084E-06 |
| DDX60 | 2.797957 | 1.53E-15 | 1.21E-12 |  | DDX60 | 1.441681 | 0.002146 | 0.02891767 |
| OAS3 | 3.987956 | 7.23E-22 | 1.51E-18 |  | OAS3 | 1.24523 | 0.003106 | 0.036508195 |
| OAS2 | 5.801791 | 7.79E-10 | 2.63E-07 |  | OAS2 | 1.886006 | 9.66E-05 | 0.003399381 |
| OASL | 4.500902 | 4.06E-05 | 0.002824 |  | OASL | 1.756854 | 0.001611 | 0.024141147 |
| XAF1 | 4.623454 | 3.11E-17 | 3.22E-14 |  | XAF1 | 1.942337 | 2.76E-05 | 0.001297371 |
| MX1 | 5.551303 | 4.97E-25 | 2.06E-21 |  | MX1 | 1.313085 | 0.001631 | 0.024283733 |

**Supplementary Table 4. Differential-expression statistics in GSE210058 and GSE159720.**

**Supplementary Table 5.** **Candidate drugs (top 10) identified from gene-drug interaction enrichment analysis** **based on the stringent 52-gene shared signature.**

| **Name** | ***P* value** | **Adj *P*-value** | **Odds Ratio** | **Combined score** |
| --- | --- | --- | --- | --- |
| suloctidil HL60 UP | 1.73E-24 | 1.66E-21 | 77.65 | 4248.77 |
| prenylamine HL60 UP | 6.24E-23 | 2.99E-20 | 229.87 | 11752.85 |
| acetohexamide PC3 UP | 2.08E-18 | 6.64E-16 | 298.02 | 12134.08 |
| 3'-Azido-3'-deoxythymidine CTD 00007047 | 4.66E-13 | 1.12E-10 | 20.05 | 569.19 |
| chlorophyllin CTD 00000324 | 1.30E-10 | 2.29E-08 | 108.28 | 2465.08 |
| prochlorperazine MCF7 UP | 1.43E-10 | 2.29E-08 | 60.69 | 1375.54 |
| terfenadine HL60 UP | 1.83E-10 | 2.51E-08 | 58.39 | 1309.11 |
| mefloquine HL60 UP | 1.11E-08 | 1.32E-06 | 88.32 | 1618.05 |
| Tetradioxin CTD 00006848 | 1.68E-08 | 1.79E-06 | 5.06 | 90.52 |
| Arsenenous acid CTD 00000922 | 8.59E-08 | 8.23E-06 | 6.55 | 106.62 |

**Supplementary Table 6. Molecular docking of mefloquine with viral proteins selected from BA-relevant viral contexts**

| **Viral context** | **Protein target** | **PDB ID** | **Best score** | **Other modes** |
| --- | --- | --- | --- | --- |
| HCMV | UL80 protease/assemblin | 8J3T | −7.2 kcal/mol | −7.1 to −6.3 |
| Rotavirus | VP1 RNA-dependent RNA polymerase | 2R7X | −6.4 kcal/mol | −6.0 to −5.3 |
